# The role of stigma and depression in the reduced adherence among young breast cancer patients in Hungary

**DOI:** 10.1186/s40359-023-01355-4

**Published:** 2023-10-09

**Authors:** Gabriella Vizin, Tamás Szekeres, Anita Juhász, Lilla Márton, Magdolna Dank, Dóra Perczel-Forintos, Róbert Urbán

**Affiliations:** 1 Institute of Psychology, Eötvös Loránd University, Izabella utca 46, Budapest, 1064 Hungary; 2 Faculty of General Medicine, Department of Clinical Psychology, Semmelweis University, Üllői út 25, Budapest, 1095 Hungary; 3 Faculty of General Medicine, Department of Surgery, Transplantation and Gastroenterology, Semmelweis University, Üllői út 78, Budapest, 1082 Hungary; 4 Faculty of General Medicine, Department of Internal Medicine and Oncology, Oncology Profile, Semmelweis University, Tömő u. 25-29, Budapest, 1st, 1083 Hungary

**Keywords:** Breast cancer, Adherence, Depression, Stigma, Body shame, Self-compassion

## Abstract

**Background:**

The main aim of our study was to investigate the role of depression, stigmatization, body shame and self-compassion in the adherence of young Hungarian breast cancer patients aged between 18 and 45 years.

**Methods:**

In a cross-sectional online survey, data were collected from 99 young breast cancer patients (BC). Participants completed self-report questionnaires on socio-demographic and cancer-specific parameters as well as psychological factors (adherence: 12-item Medication Adherence Scale; depression: Hospital Anxiety and Depression Scale; stigmatization: Stigma Scale for Chronic Illnesses; body shame: Experience of Shame Scale; self-compassion: Self-Compassion Scale). We tested the predictors and mediators of adherence using hierarchical regression, mediation and moderation analysis among BC patients.

**Results:**

We found that adherence was significantly associated with body shame and stigmatization in our BC sample. In addition, stigmatization alone was a significant predictor of lower adherence. Finally, in mediation models, where body shame was a mediator, we found a significant direct effect between stigma and adherence, in other words body shame had a significant mediating effect between these variables. According to our moderation analysis, self-compassion as a significant moderator acts as a protective factor in the linear relationship between stigma and lower adherence.

**Conclusions:**

Our results highlight the importance of stigma and body shame in the development of adherence in oncological care among young Hungarian BC patients aged between 18 and 45 years. Assessment of stigma, body shame, self-compassion, and the improvement of the availability of evidence-based psychological interventions may increase the adherence of young Hungarian BC patients, leading to more favourable rates of survival.

## Introduction

The most common type of cancer among women is breast cancer (BC) [1]. Despite survival rates show an improving trend in recent decades due to major advances in cancer treatment [1, 2], still Hungary has the highest BC mortality rate in the EU [1]. Although BC mortality rate is higher in women older than 45 years, BC incidence (approx. 850 young BC patients in Hungary per year) and mortality are higher in younger women (18–45 years) than in other cancers [1]. It is a well-known fact that screening, and adherence to treatment contribute to increased BC survival [3–5], however, the lack of recommended screening until the age of 45, the lack of self-examination practices among young women, and non-adherence to treatment by patients may play a role in the occurrence of higher mortality rates among young breast cancer patients in Hungary [6]. Examining these factors and developing effective psychological interventions to improve them appear to be crucial to achieve higher survival rates [6, 7].

Focusing on adherence, between one-third to one-half of women with BC stopped taking medication early, and compared with women with breast cancer aged 45 years or older, those younger than 45 years were more likely to be nonadherent [8–10]. Adherence may be influenced by several treatment-related factors, and it has been recognized that adherence was effected by depression [11, 12] and stigmatization [13].

The prevalence of a depressive episode is more than five times higher for BC patients than for the general population [14]. Although BC correlates with distress and mood disturbances at all ages [15], the high risk of losing fertility [16] and the impact of cancer treatments to body image and sexuality may lead to increased risk of developing mood disorders, such as depression in premenopausal BC patients [17, 18], which can lead to poorer adherence in this population [19, 20].

Stigma also fundamentally determines health behaviors [21], which includes lower uptake of screening for disease, adherence to medication, and poorer health outcomes for multiple conditions among cancer patients [22]. However, cancer was a severe stigma that had eased in recent decades [23, 24], studies have found that BC survivors still experience the impact of stigmatization [25]. Although visibility (such as alopecia or scars) and peril (life threatening diagnosis) as external stigmas [26] significantly correlated with poorer health behavior [27], with regard to the most significant negative consequences, studies emphasize importance of internalized stigma or self-stigma [26]. Through the process of internalization, stigma becomes an internal cognitive content associated with experience of self-blame and feelings of shame [28]. Internalized stigma correlates with depression and lower medication adherence among HIV patients and cancer patients [14], but factors that may be thought to underlie these relationships, such as bodily shame or self-compassion, have not been studied before in young women with BC.

Shame is a negative self-conscious emotion that includes worthlessness, helplessness, intent to hide, and self-blame [29]. Shame and self-blame have been linked to higher levels of distress and lower psychological adjustment among women with BC [30]. One of the shame factor is the body-related shame [31]. Although we clearly know that young BC patients have body-image disturbances which is correlated to body shame [17, 18, 32], and body shame correlates with body-related stigma among obese people [33], but we have not found research on this association in young BC patients yet.

The ”antidote” of shame and stigma is self-compassion [34, 35]. According to the definition of self-compassion, it means looking at ourselves kindly, without judgement for personal suffering or perceived failures [35]. Self-compassion may buffer the negative effects of stigma [34], and higher levels of self-compassion have a potentially protective effect in women with BC at risk for body image disturbance [18]. However, we have not found any research, which would have investigated correlations between self-compassion and stigma in young BC population.

Based on previous assumptions, the first aim of our research was to investigate the relationship between adherence, depression, stigma, body-related shame and self-compassion in young BC patients. Although the relationship between depression, stigma and adherence have been investigated before [6, 7, 14, 19, 20], the exact nature of the relationship between these variables and the exploration of other underlying factors (such as body shame and self-compassion) have been recognized necessary, in order to increase the survival rate of young BC patients aged 18–45.

In particular, we hypothesized that (1) there is a positive correlation between depression, stigma, and body shame, and negative correlation between adherence, depression, stigma and body shame. In addition, we supposed that (2) the adherence is influenced by depression and stigma as predictors in our sample. Finally, we presumed that (3) these relationships are mediated by body shame and self-compassion, and (4) self-compassion has a moderating effect between predictors and adherence.

## Methods

### Participants

We collected data from 99 participants in Hungary in a cross-sectional online study using a set of standardized questionnaires. The inclusion criteria were the diagnosis of BC, non-metastatic cancer-status, receiving active treatment (operation, chemotherapy, radiotherapy, endocrine therapy), female sex and age from 18 to 45 years. Exclusion criteria were the metastatic cancer-status, self-reported psychiatric disorder (such as depression, anxiety, etc.) past or present, and higher age than 45 years. The sample included 99 women, but five participants were excluded because of the exclusion criteria. Finally, the sample contained 94 women (mean age = 38.76; SD = 6.48).

### Procedure

The sample was collected at the Semmelweis University Department of Oncology and from breast cancer-specific social media groups (Hungarian Facebook groups of patients with breast cancer and breast cancer survivals) in Hungary. We collected data online from December 2018 to December 2020 from the non-metastatic BC population aged 18–45 in Hungary. In all cases, we recruited the patients using a flyer. The flyer included the title of this study, aims and short description of the study, and the link of online survey. The participants gave informed consent online in all cases. During the data management, we checked for duplicate filling data, but did not find any.

### Measures

#### Demographics and disease-related information

Descriptive data of the sample are presented in Table 1. Descriptive data were collected through a demographic questionnaire, which consisted of questions about age, sex (assigned female at birth = AFAB), marital status, education level, and short history of somatic and psychic events. Information of the disease included date of BC diagnosis and types of previous and current oncological treatments (operation, chemotherapy, radiotherapy, endocrine therapy).

#### Adherence

Ueno et al. [36] developed the 12-item Medication Adherence Scale (MAS) to measure the comprehensive concept of medication adherence. The scale applies to all medicines prescribed by a doctor in the last six months in relation to cancer. “Medication” includes medicine administered orally, injections, ointments, medicated patches, and inhalants, including endocrine therapy, chemotherapy and drugs for the treatment of side effects in this period. MAS includes four subscales, each subscale containing three items that measure (1) medication compliance (e.g. “Over the past 3 weeks, I have taken the prescribed daily dosage of my medication.”), (2) collaboration with healthcare providers (e.g. “I feel comfortable asking my healthcare provider about my medication.”), (3) willingness to access and use information about medication (e.g. “I understand both the effects and the side effects of my medication.”), and (4) acceptance to take medication and the fitness of taking medication to the patient’s lifestyle (e.g. “Taking medication is part of my everyday life, just like eating or brushing my teeth.”). These items are rated on a five-point scale ranging from 1 (“never”) to 5 (“always”). Higher scores indicate higher medication adherence. MAS is a reliable scale for measuring medication adherence in many diseases [14, 37] such as cancer [38]. The English version of MAS was translated to Hungarian by two researchers (one of them is the first author of this publication), then an independent researcher translated back to English. During the translation process, the steps of Beaton’s protocol were followed [39]. Internal consistency of MAS is applicable in the present study (Cronbach alpha = 0.87).

#### Depression

Hospital Anxiety and Depression Scale (HADS) is a scale of two combined 7 items, one targeting anxiety (HADS-A) and the other targeting depression (HADS-D) [40]. Each item is rated on a four-point scale with a maximum score of 21 for anxiety and depression scales each. HADS is a widely used scale in oncological setting and is a reliable screening instrument [41]. Muszbek and her colleagues [14] translated HADS to Hungarian language, and they found that both reliability and validity test results confirmed HADS as a suitable scale for measuring depression and anxiety in cancer patients. In our present study, we used the depression subscale of HADS to measure depression (Cronbach alpha = 0.85).

#### Stigma

*Stigma Scale for Chronic Illnesses (SSCI-8)* [42] is a valid and reliable 8-item scale for measuring external (enacted) and internalized stigma. Each item (e. g. „Because of my illness, some people seemed uncomfortable with me.” or „I felt embarrassed about my illness.”) is rated on a 5-point Likert scale from 1 (never) to 5 (always). Total scores range from 8 to 40, with higher scores indicating higher levels of stigma. SSCI-8 is a suitable scale measuring stigma in BC patients [43]. The Hungarian version was developed by Szőcs and her colleagues [44]. Internal consistency of SSCI-8 is 0.92 in the present study.

#### Body shame

Experience of Shame Scale (ESS) was developed to measure three domains of chronic shame: body shame (e.g., “Have you ever felt ashamed because of your body or a specific body part?”), characterological shame (e.g., “Have you ever felt ashamed because of your own habits?”), and behavioral shame (e.g., “Have you ever felt ashamed for doing something wrong?”) [31]. ESS consists of 25 items rated on a 4-point scale from 1 (not at all) to 4 (very much). Based on the validation study of the Hungarian version of this questionnaire, ESS is a valid and reliable scale of chronic shame in Hungarian clinical and healthy samples [45]. We used body shame factor from ESS to measure body-related shame in present study (Cronbach alpha = 0.87).

#### Self-compassion

Self-Compassion Scale (SCS) was developed by Kristin Neff [35] and is able to reliably assess the level of self-compassion. This scale includes items that measure how often people respond to suffering with self-kindness (e.g., “I try to be loving toward myself when I’m feeling emotional pain”), self-judgment (e.g., “I’m disapproving and judgmental about my own flaws and inadequacies”), common humanity (e.g., “I try to see my failings as part of the human condition”), isolation (e.g., “When I think about my inadequacies, it tends to make me feel more separate and cut off from the rest of the world”), mindfulness (e.g., “When something painful happens I try to take a balanced view of the situation”), and over-identification (e.g., “When I’m feeling down I tend to obsess and fixate on everything that’s wrong”). Responses are rated on a 5-point scale from “Almost Never” to “Almost Always.”. Hungarian version was developed by Tóth-Király and his colleagues [46]. Internal consistency of SCS is 0.92 in the present study.

### Statistical analysis

First, to estimate the number of sample size, we used Cohen’s [47] definition of an expected value below 0.2, i.e. a maximum 20% chance of missing an existing effect. Based on the estimated effect and noise magnitude, we estimated the minimum number of items needed to have a statistical power of 80% at the 5% significance level [48]. Accordingly, at a 5% significance level and β < 20%, with 0.60 ≤ r - strong effect, a minimum variable of n = 19 participants (n = 95 for 5 variables) were required. As a second step, the basic statistical characteristics of the sample were calculated, and then we calculated the Cronbach alpha values of the questionnaires used in the study. The statistics were published describing the measuring devices used in the study and to test the differences between the samples in the framework of two-variable analyses. Third, an evaluation was conducted to determine the relationship between our variables based on the Pearson correlation matrix of previously validated questionnaires related to adherence and depression. Cohen’s definition was applied to interpret the values of correlation coefficients. In the next step, the explanatory power of the variables examined in relation to adherence were studied with hierarchical regression. Based on the outlier labelling rule, no one-dimensional outlier was found [47, 49]. To filter multidimensional outliers, we calculated Mahalanobis distance and checked the data at the following criterion level: Mahalanobis distance p < 0.001 at 40.47 [50], Cook distance 1 [51]. These results indicate that there is no multidimensional outlier in the data. Based on tests the condition of collinearity can be verified, so that there is neither collinearity nor multicollinearity among variables. The high independence of predictors allows us to clearly interpret their effect in regression. The data is met with the condition of independence of residual errors, the Durbin-Watson value = 1,617 [52]. Thereafter, the relationship between stigmatization, shame and adherence were tested. We examined the effect of the predictor and output, predictor and mediator, predictor and mediator output variable, as well as the strength of the coefficient of the predictor in hierarchical regression compared to simple regression. Finally, to test the applicability of self-compassion, the following model was tested: the effect of self-compassion as a moderating variable on the relationship between stigma and adherence in multiple regressions. Statistical analyses were carried out using SPSS 24 and JASP programs.

## Results

### Descriptive statistics

Based on the demographic results, the mean age was 38.88 years (SD = 6.56), the average number of years spent in the education was 16.02 (SD = 3.29). 69% of the participants (n = 65) are married or in relationship, 64% of the patients (n = 61) have children. Cancer related variables show that the time since diagnosis is 10.69 months (SD = 11.16), and operation was performed in 69 cases (73%). The type of operation is more likely to be mastectomy (42 cases), and a smaller proportion of breast-conserving surgery was performed (27 cases). Twenty-six patients had been still receiving anti-cancer treatment (chemotherapy and/or radiotherapy and/or endocrine therapy). The results of the descriptive statistics for study variables are presented in Table 1.

**Table 1:** Descriptive statistics and cancer related variables. M = mean, SD = standard deviation

VARIABLES		No. of participants (N) = 94
	**Demographic variables (Mean, SD, years)**	
Age (at registration): Range (min-max)		38.88 (6.56) 32.32–45.44 years
Level of education:		16.02 (3.29)
	**Relationship status (N, %)**	
with partner		65 (65)
without partner		37 (36)
	**No. of children**	
0		33 (34)
1–4		61 (66)
	**Cancer related variables**	
Time since diagnosis: Mean (SD), months		10.69 (11.16)
Operation has been performed (N, %)		69 (73)
Time since operation: Mean (SD) months		6.22 (6.29)
	**Type of operation**	
Mastectomy (N, %)		42 (60)
Breast-conserving surgery (N, %)		27 (40)
	**Current treatment method**	
Chemotherapy and/or radiotherapy and/or endocrine therapy (N, %)		26 (24)

**Table 2:** Correlations for study variables. Notes: N = 94, *p < 0.05. **p < 0.01. ***p < 0.001, M = mean, SD = standard deviation

Variable	M	SD	Age	Education	Adherence	Depression	Stigma	Body shame
Age (years)	38.88	6.56						
Level of education (years)	16.02	3.29	-0.083					
Adherence	50.79	8.73	0.125	-0.238*				
Depression	6.73	4.14	-0.039	0.038	-0.204			
Stigma	17.38	7.41	-0.280**	0.074	-0.361***	0.524***		
Body shame	9.91	4.23	-0.376***	0.112	-0.226*	0.469***	0.614***	
Self-compassion	76.35	19.21	0.253*	0.045	0.101	-0.493***	-0.371**	-0.544***

### Correlations of psychological variables in young breast cancer sample

Stigma and body shame show weak negative coexistence with age, and self-compassion shows positive, weak coexistence with age. In addition, adherence levels areweak, showingnegative coexistence with body shame, as well as it showsnegativeassociation, with moderate strength in associationwith stigma. However, depression has a significant moderately positive correlation with stigma and body shame, but a negative correlation with self-compassion (see Table 2.).

### Examination of the explanatory power of the studied variables for adherence

Using hierarchical regression, we analyzed the extent to which adherence can be explained by depression, body aspects of shame, stigma, and self-compassion in individuals within breast cancer group, compared to healthy controlsample.

In the first level of hierarchical regression, depression, in the second level, shame, stigma, and self-acceptance were included in the model (ENTER). The characteristics of the variables entering the regression shall be determined in accordance with the procedure explained inTable 3. While the first model explains only 8% of the variation in adherence, the second model explains 26.9% of the output variable and the first explanatory force model: F(4.68) = 1,331 p < 0.05.However, the data in Table 3. indicate that of the coefficients, only the effect of stigma is significant, and that by including it in the model, the explanatory power of depression is also decreased and does notadd significantly to the explained variance of adherence.

**Table 3:** Examination of the explanatory power of the variables examined for adherence by hierarchical regression

	β	SE(β)	β	t	p
Model 1					
*Constant*	53.704	1.747		30.749	< 0.001
*Depression*	-0.419	0.220	-0.199	-1.903	< 0.045
Model 2					
*Constant*	56.535	2.969		19.045	< 0.001
*Depression*	0.099	0.329	0.044	0.302	0.764
*Stigma*	-0.352	0.179	-0.286	-1.964	< 0.043
*Body shame*	-0.046	0.354	-0.021	-0.130	0.897
*Self-compassion*	-0.011	0.078	-0.024	-0.144	0.886

### Mediation and moderation analyses

**Fig. 1:**
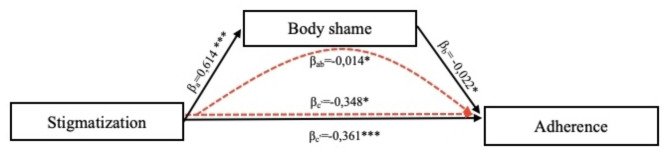
The relationship of stigma through body shame to adherence *Note*: * p < 0.05; *** p < 0.001

Following an examination of the explanatory power of adherence, it was examined that the relationship between stigma and adherence is not direct, but indirect, acting through a mediating factor, which is body shame/self-compassion. The full model is illustrated in the following Fig. 1; Table 4. Our hypothesis about the indirect effect of body shame has been fully confirmed. In other words, the relationship between stigmatization and adherence is not linear, but it indirectly affects cooperation with treatment through body shame of individuals with breast cancer.

**Table 4:** Mediation models for examining the role of body shame as mediators between stigma and adherence

x		y	β	SE(B)	t	p	LLCI	ULCI
*Stigmatization (total)*		*Adherence*	-0.361	0.100	-3.630	< 0.001	-0.561	-0.164
*Stigmatization*		*Body shame*	0.614	0.083	7.420	< 0.001	0.452	0.783
*Stigma*		*Adherence*	-0.348	0.126	-2.777	0.041	-0.599	-0.099
*Body shame (direct)*			-0.022	0.126	-0.177	0.045	-0.270	-0.226
*Stigma through body shame (indirect)*	*Adherence*		-0.014	0.076		0.046	-0.162	-0.135

*Note*: LLCI & ULCI: Lower and upper levels of the 95% confidence interval.

Mediation analysis revealed a significant linear relationship between stigmatization and reduced adherence, with body shame playing a mediating role. We next examined whether the level of self-compassion moderates this relationship. No one-dimensional outlier excluding the test was found in the data based on the outlier labelling rule [49].

**Fig. 2:**
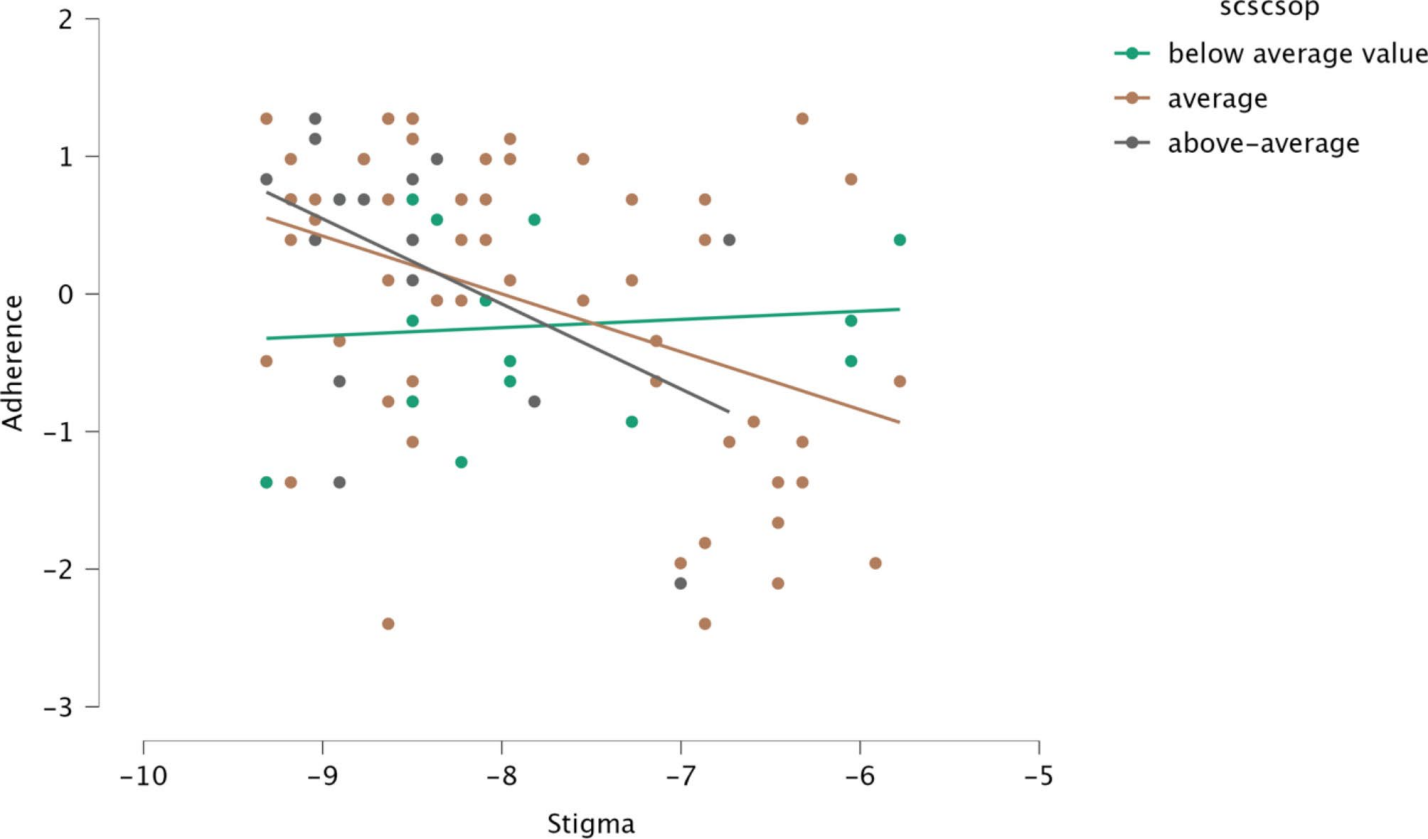
The moderating effect of self-compassion in the association of stigmatization with reduced adherence

In our moderation model, stigmatization explained 14% of the variance in adherence (R2Adj = 0.141), and the model is significant, F(1,92) = 7.570 p < 0.001. The effect of interaction (stigma x self-compassion) is also significant (p <. 001.). Including the interaction in the model increased the explained variance by 0.03%, and this is a significant increase F(1, 91) = 3.380 p < 0.005. All these relationships are illustrated in Fig. 2 below.

## Discussion

The main goal of our research was to explore the association between adherence and depression, stigmatization, body shame and self-compassion among young Hungarian breast cancer patients. Firstly, we investigated the relationship between adherence, depression, stigma, body shame and self-compassion using correlation analysis. Our results suggested that there had been significant positive correlations between stigma, body shame and depression, and a negative significant association between self-compassion and depression. Previous research found similar results among these factors in BC patients [18, 53] as well as in young BC patients [14, 32]. Nevertheless, it is a surprising result that adherence is significantly associated with body shame and stigma instead of depression in our Hungarian sample. This result contradicts previous findings that depression is a crucial factor in adherence [19], especially in young BC patients [20]. However, this result highlights the importance of disease-related stigma in adherence [21, 22].

Our main results came from a hierarchical regression model in BC sample. In the first model, depression was a significant predictor of lower levels of adherence. However, in the second model, adding factors of body shame, stigma, and self-compassion to the model, the effect of depression disappeared, and stigma alone became a significant predictor of lower levels of adherence. This is a very surprising result, as based on the results of previous studies, we also expected the effect of depression in this model [19, 20]. Although our results contradict previous results in BC sample [19, 20], there are a number of studies among HIV-infected people that highlight the role of stigma in adherence. Sweeney and Vanable [54], in their systematic review of HIV-related stigma and adherence, summarize that stigma-concept and factors are vague in studies and that questionnaires are inadequate in some cases, but stigma may increase vulnerability to mental health problems, which in turn interferes with self-care activities that support survival, such as adherence. According to a recent meta-analysis, stigma may negatively influence help-seeking behavior and confrontational coping as survival behaviors, however, there is a positive association between stigma and depression among BC patients [14]. Although our correlation analysis results showed similar relationship between these variables but the level of perceived stigmatization can be greater among young BC patients [14], which may determine its stronger effect on adherence instead of depression. Similar to this assumption, our results suggest that, instead of depression, higher stigma predicts poorer adherence in young BC patients in Hungary.

Finally, we examined body shame as a mediator and self-compassion as a moderator between stigma and adherence in a young BC sample. According to our results, there is a significant direct effect between stigma and adherence, and body shame has a significant mediating effect between these variables. Our results suggest that stigma may be a stronger predictor of adherence in cases of higher body shame and self-blame. Although body image disturbances are well known among BC patients [14, 55], we found that the effect of body shame on adherence has not been investigated. The role of body shame is a new finding among BC patients, but referring back to the HIV studies, Sweeney and Vanable’s [54] conclusion includes this possible pathway between stigma and adherence, especially if we rely on our previous knowledge of the significant relationship between body shame and mental health difficulties [56]. On the other hand, based on Beck’s cognitive theory [57] and Corrigan’s theory of self-stigma [26], the path between awareness and internalization of stigma may be influenced by early shameful experiences (e.g., repeated stigmatization, bullying or body humiliation previously). This means that among BC patients, the diagnosis of BC may be associated with negative automatic thoughts (such as: “I am stigmatized”) that can lead to body shame, and poorer self-care activities. According to our moderation analysis (Fig. 2), we found that stigma leads to lower adherence to a lesser extent in individuals with average and above-average self-compassion than in individuals with below-average self-compassion. In other words, self-compassion is a protective factor in the linear relationship between stigma and lower adherence. This implies that self-compassion may be an antidote to shame and stigma among BC patients [34, 35] and may support self-care activities such as adherence. Self-compassion skills can be developed through focused psychological intervention [18], which is a promising way to increase the survival rate of young Hungarian BC patients.

### Study limitations

Our research was not without limitations. Due to the cross-sectional nature of our study, it was impossible to assess causal relationships between the variables. In addition, recall bias might have influenced the responses on the online self-reported questionnaires. Furthermore, our BC sample was well defined but small, thus conclusions about stigma and adherence in Hungarian young BC patients should be treated with caution. To assess adherence, participants completed the MAS-12 scale, but we need further investigation about psychometric characteristics of this scale in Hungarian cancer samples. Our results regarding adherence may also be affected by convenience sampling. The voluntary online survey influenced the characteristics of the participants, so that our sample was not representative. Based on these mentioned limitations, we plan to repeat the study to better understand the potential effect of adherence, stigma and depression, as well as the impact of demographic and disease-related variables on a comprehensive Hungarian cancer sample.

### Clinical implications

Our results highlight that in Hungary, adherence in young breast cancer patients is influenced by stigma, rather than depression, especially if the patient is more prone to bodily shame. In the Hungarian psycho-oncological care, cognitive behavioural therapy (CBT) and self-compassion-focused methods are still underrepresented, although these are evidence-based approaches in both depression and stigma (or shame) in cancer patients [58, 59]. Promising results have been obtained in this population in the behavioral activation, problem solving, cognitive restructuring, gratitude diary, and development of self-compassion [14, 18, 60–64]. In the future, comprehensive, complex, time-limited CBT-based interventions are needed for oncological patients to better adherence and reduced mortality in Hungary.

## Conclusion

Our results highlight the importance of stigma and body shame in the development of adherence in oncological care among young Hungarian breast cancer patients. After the shocking diagnosis of breast cancer, assessing stigma and body shame, and improving the availability of evidence-based psychological interventions may increase the adherence and survival rates of Hungarian breast cancer patients.

## Data Availability

The data that support the findings of this study are available from the corresponding author upon reasonable request.
